# MIAT: An important long non-coding RNA regulating cardiovascular and cerebrovascular diseases

**DOI:** 10.3389/fcvm.2026.1791657

**Published:** 2026-07-13

**Authors:** Yuxin Pan, Wusheng Yu, Yantao Han, Yingchun Shao

**Affiliations:** 1 School of Basic Medicine, Qingdao University, Qingdao, China; 2 Department of Internal Medicine, Qingdao Third People's Hospital Affiliated to Qingdao University, Qingdao, China; 3 Department of Pharmacy, Qingdao Municipal Hospital, Qingdao, China

**Keywords:** biomarker, cardiovascular diseases, cerebrovascular diseases, long non-coding RNA, MIAT

## Abstract

Cardiovascular and cerebrovascular diseases remain the leading cause of death worldwide. Myocardial infarction-associated transcript (MIAT), a type of long non-coding RNA, has been reported to exert regulatory functions across multiple diseases in studies, and may serve as a promising therapeutic candidate in certain pathological conditions. This review provides a comprehensive analysis of the basic characteristics of MIAT, the latest research advancements, its involvement in cardiovascular and cerebrovascular diseases, and its potential as a biomarker and therapeutic target. The objective is to summarize the current understanding and mechanisms of MIAT, offering further scientific evidence to support its role in the treatment of cardiovascular and cerebrovascular diseases associated with MIAT. Additionally, this review explores the challenges and practical considerations for the clinical application of MIAT, aiming to provide new perspectives on its clinical translation.

## 1 Introduction

Cardiovascular and cerebrovascular diseases encompass a range of ischemic or hemorrhagic conditions affecting the heart, brain, and other tissues throughout the body, typically driven by factors such as hyperlipidemia, blood viscosity, atherosclerosis (Ath), and hypertension (1–3). These diseases remain the leading cause of death worldwide (4–6).

Non-coding RNAs have emerged as key regulatory molecules in gene expression, playing pivotal roles in various physiological and pathological processes (7–9). Long non-coding RNAs (lncRNAs) are a subclass of non-coding RNAs that regulate gene expression by interacting with chromatin regulators and modulating RNA activity, thereby influencing cellular responses (10, 11). Myocardial infarction-associated transcript (MIAT), a specific lncRNA, has garnered significant attention due to its involvement in various diseases beyond myocardial infarction (MI). Research indicates that MIAT is implicated in ischemic stroke (12), hepatocellular carcinoma (13), vascular eye diseases (14), squamous cell carcinoma (15), uterine fibroids (16), diabetic retinopathy (17), ovarian cancer (18), osteoporosis (19), and acute kidney injury (20), among others.

Although no clinical trials targeting the lncRNA MIAT have been registered to date, reported aberrant expression of MIAT in multiple disease contexts render it a promising candidate for future translational research.However, despite these advances, few reviews have summarized the roles of MIAT in cardiovascular diseases, but no comprehensive review has yet integrated both cardiovascular and cerebrovascular functions of MIAT, nor provided an updated synthesis of recent advances up to 2025 with a specific focus on translational applications.Compared with prior reviews focusing only on cardiovascular disease, the present work offers three key advances: (1) it integrates both cardiovascular and cerebrovascular pathologies, providing a unified perspective on MIAT's role in vascular biology; (2) it includes literature published up to 2025, incorporating newly identified mechanisms, biomarker studies, and preclinical therapeutic data; and (3) it explicitly discusses the translational potential of MIAT as a diagnostic marker and therapeutic target, with a focus on current challenges and future directions for clinical application.

This review systematically details the basic characteristics of MIAT, summarizes key research advances, explores its role in cardiovascular and cerebrovascular diseases, and evaluates its potential as a biomarker and therapeutic target. It aims to provide a thorough overview of MIAT's current research status, mechanisms, and clinical applications, thereby offering valuable insights into its potential for clinical translation.

## 2 Basic characteristics of MIAT

MIAT, also known as Gomafu or RNCR2 (21, 22), was first identified as a complementary DNA and consists of five exons (23). Subsequent studies revealed that MIAT is a lncRNA with transcripts greater than 200 nucleotides, expressed across various cells and tissues (24). Located on chromosome 22q12.1, MIAT has four distinct splicing variants (23, 25), which play different roles in various physiological and pathological contexts (26–28).

Genome-wide association studies have identified single nucleotide polymorphisms at six loci of MIAT, linking these variations to the incidence of myocardial infarction in humans (29, 30). MIAT is predominantly localized in the nucleus, where it participates in the pathophysiology of the heart by influencing ribosome biogenesis and translational reprogramming (10, 31, 32). It also plays a pivotal role in gene expression regulation, with its specific chromosomal location potentially interacting with nearby genomic regions to affect the expression of adjacent genes (25, 33).

It is worth noting that MIAT exhibits dynamic subcellular localization that is cell-type dependent and context-specific, with accumulating evidence supporting its presence in the cytoplasm under certain conditions. In podocytes, RNA-FISH demonstrates MIAT localization in both the cytoplasm and nucleus (34), while co-localization with microRNAs (miRNAs) in the cytoplasm has been confirmed in vascular smooth muscle cells and hepatic stellate cells (35). Additionally, in rat hippocampal neurons, RNA-FISH demonstrates a co-localization of MIAT and the cytoplasms (36). However, MIAT is not uniformly cytoplasmic; in cardiomyocytes, it predominantly localizes to the nucleus (37), and databases such as LncSpA annotate it as nuclear or lamina-associated. This cell- and environment-dependent distribution underlies a “location-function” paradigm: nuclear MIAT primarily acts as a transcriptional regulator (30, 37, 38), whereas cytoplasmic MIAT functions as a competing endogenous RNA (ceRNA) by sponging miRNAs to modulate gene expression (35, 39). The precise mechanisms governing MIAT export from the nucleus to the cytoplasm remain incompletely understood, but this dynamic localization is likely a critical regulatory node for its diverse roles in physiology and disease.

Beyond its dynamic subcellular localization, MIAT also exhibits transcript diversity through alternative splicing, with several isoforms that contribute to its functional complexity. Current evidence supporting the functional roles of MIAT splicing isoforms mainly focuses on splicing regulation (37, 40, 41) and disease risk association (23). In terms of splicing regulation, MIAT can recruit RBFOX2 (40) or directly bind to the splicing factor SF1 (37), thereby affecting the alternative splicing of key genes such as Mcl-1 (40), CGRP (37), and Wnt7b (41), and playing an important role in neurodevelopment, spinal cord injury repair, and leukemogenesis. Regarding disease risk, a risk haplotype consisting of single nucleotide polymorphisms (SNPs) located in intron 1 and exons 3 and 5 of the MIAT gene is significantly associated with genetic susceptibility to myocardial infarction (23), suggesting that splicing or expression variations of MIAT itself have clear pathological effects. In summary, MIAT not only serves as a molecular platform for regulating the splicing of other genes, but also the structural changes and expression levels of its own isoforms are closely related to the development and progression of various diseases.

Moreover, MIAT interacts with a variety of molecules to exert its biological functions. For instance, during the pathological processes of the heart, MIAT binds to nucleolin, leading to a reduction in MIAT levels and subsequently inhibiting myocardial hypertrophy. In MIAT knockout mice or cultured cardiomyocytes, pathological hypertrophy is significantly alleviated, accompanied by impaired ribosomal RNA biogenesis and processing, as well as reduced protein synthesis (31). In MI, MIAT interacts with FUS, which helps promote cardiac cell survival under oxidative stress (29). Additionally, in brain cancer, MIAT regulates the proliferation, migration, and metastasis of brain cancer cells by modulating the Nanog/Sox2/let-7a-5p/miR-29b-3p axis (42). The basic characteristics of MIAT are summarized in Table 1.

**Table 1:** Basic characteristics of MIAT.

Feature	Description	References
Chromosomal location	Chromosome 22q12.1	(23, 25)
Splicing variants	Four distinct splicing variants with diverse roles in physiological and pathological processes	(23, 25–28)
SNPs	Six susceptibility loci associated with myocardial infarction risk	(29, 30)
Subcellular Localization	Predominantly nuclear	(10, 31, 32, 34–36)
	Presence in the cytoplasm under certain conditions	
Key interactions	Binds nucleolin, FUS and mitochondrial translocator protein	(29, 31, 43, 44)
	Functions as a miRNA sponge to modulate downstream gene expression	
Functional domains	Acts as a ceRNA and protein stabilizer; regulates redox homeostasis	(43, 45, 46)

## 3 Evolutionary Conservation of MIAT

Current evidence indicates that MIAT possesses a highly conserved sequence in humans. Bioinformatics analyses have identified clear homologous regions between human MIAT and those of rat and mouse (47) (e.g., human nucleotides 6,834–6,920 correspond to rat 5,705–5,791 and mouse 6,101–6,187). This conservation can be traced back to amphibians (26), suggesting that MIAT has been under selective pressure during the evolution of placental mammals and even more broadly across vertebrates. The sequence conservation is directly validated by the observation that siRNAs designed against these conserved targets achieve effective gene silencing in human, rat, and mouse cardiomyocytes (47). Furthermore, the expression pattern of MIAT is also conserved; its expression can be detected in specific retinal layers (such as the retinal pigment epithelium) in humans, rats, and mice (26). Owing to its high conservation in mammals, MIAT has been considered a potential diagnostic marker and therapeutic target for various diseases (48–50). In summary, MIAT exhibits remarkable evolutionary conservation from its nucleotide sequence to its expression and function, which underlies its core biological roles across species.

## 4 Main progress in the research of MIAT

**Figure 1:**
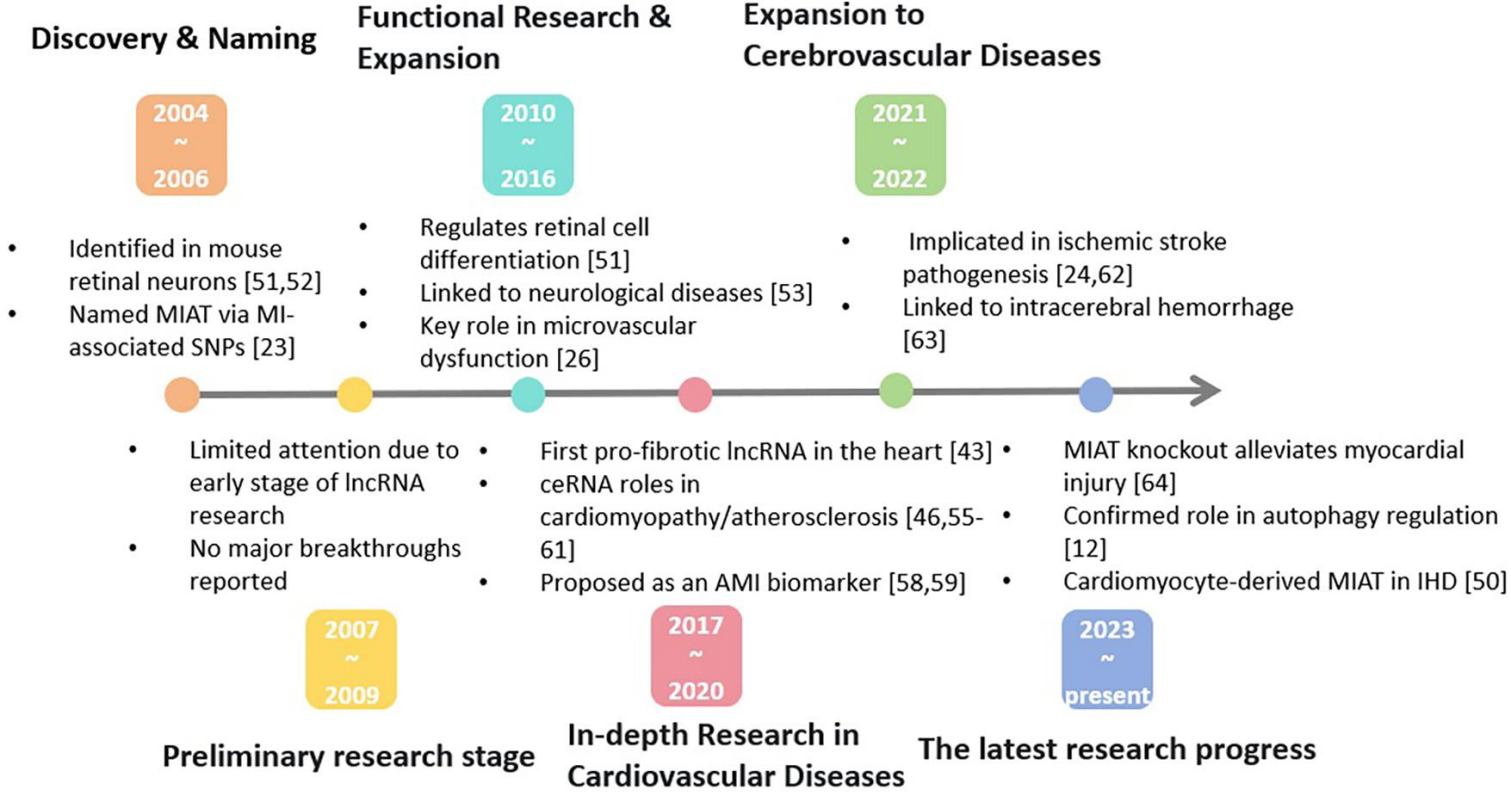
The main progress in the research of MIAT. This figure depicts a chronological timeline illustrating the key stages of MIAT research. It tracks the timeline from its initial discovery and naming in 2004–2006, through preliminary and functional studies, to its in-depth research in cardiovascular and cerebrovascular diseases, culminating in the latest breakthroughs from 2023 to the present.

MIAT was first identified in 2004 as a non-coding RNA expressed in a specific subset of neurons in the mouse retina (51, 52). In 2006, Nobuaki et al. demonstrated that SNPs in the human homolog of MIAT were linked to an increased risk of MI, leading to its designation as the MIAT (23). Between 2007 and 2009, research on lncRNAs was in its early stages, and MIAT, as a member of this group, had not garnered widespread attention. However, since 2010, MIAT has gained increasing recognition in scientific research. In 2010, it was shown that MIAT plays a role in regulating retinal cell differentiation and the pluripotency of embryonic stem cells (51). In 2014, Barry et al. highlighted that MIAT dysregulation could contribute to neurological diseases, such as schizophrenia (53). In 2015, MIAT was identified as a key regulator of microvascular dysfunction (26). In 2016, MIAT was found to be selectively upregulated in neuroendocrine prostate cancer (54). Since then, research on MIAT has expanded, revealing its involvement in various diseases. This article focuses on the research progress of MIAT in cardiovascular and cerebrovascular diseases. In 2017, MIAT was identified as the first pro-fibrotic lncRNA in the heart (43), with evidence suggesting its involvement in the pathogenesis of diabetic cardiomyopathy as a ceRNA (55), and a significant association with Ath (56). By 2018, studies showed MIAT's critical role in both Ath (46) and cardiac hypertrophy (57). In 2019, MIAT was proposed as a potential novel biomarker for acute myocardial infarction (AMI) diagnosis (58, 59). In 2020, further research revealed that MIAT regulates heart disease through its function as a ceRNA, sponging miR-133a-3p (60) and miR-214-3p (61). In 2021, the role of MIAT in cerebrovascular diseases gained attention, with studies showing MIAT's involvement in promoting autophagy and apoptosis in nerve cells during ischemic stroke (24), as well as impairing neurological function in ischemic stroke (62). In 2022, MIAT was identified as a potential player in the pathogenesis of intracerebral hemorrhage (63). In 2023, it was found that MIAT knockout alleviates myocardial cell damage induced by pirarubicin (64). By 2024, MIAT was recognized as a key regulator of autophagy in various diseases, including ischemic stroke (12). In 2025, Hayasaka et al. clarified the critical pathological role of cardiomyocyte-derived MIAT in ischemic heart disease (50), providing a solid experimental foundation for developing therapeutic strategies targeting MIAT. The above research progress of MIAT is summarized in Figure 1. These findings highlight MIAT's significant regulatory role in cardiovascular and cerebrovascular diseases, underscoring its potential as a therapeutic target in these conditions.

## 5 The role of MIAT in cardiovascular and cerebrovascular diseases

### 5.1 MIAT and MI

**Figure 2:**
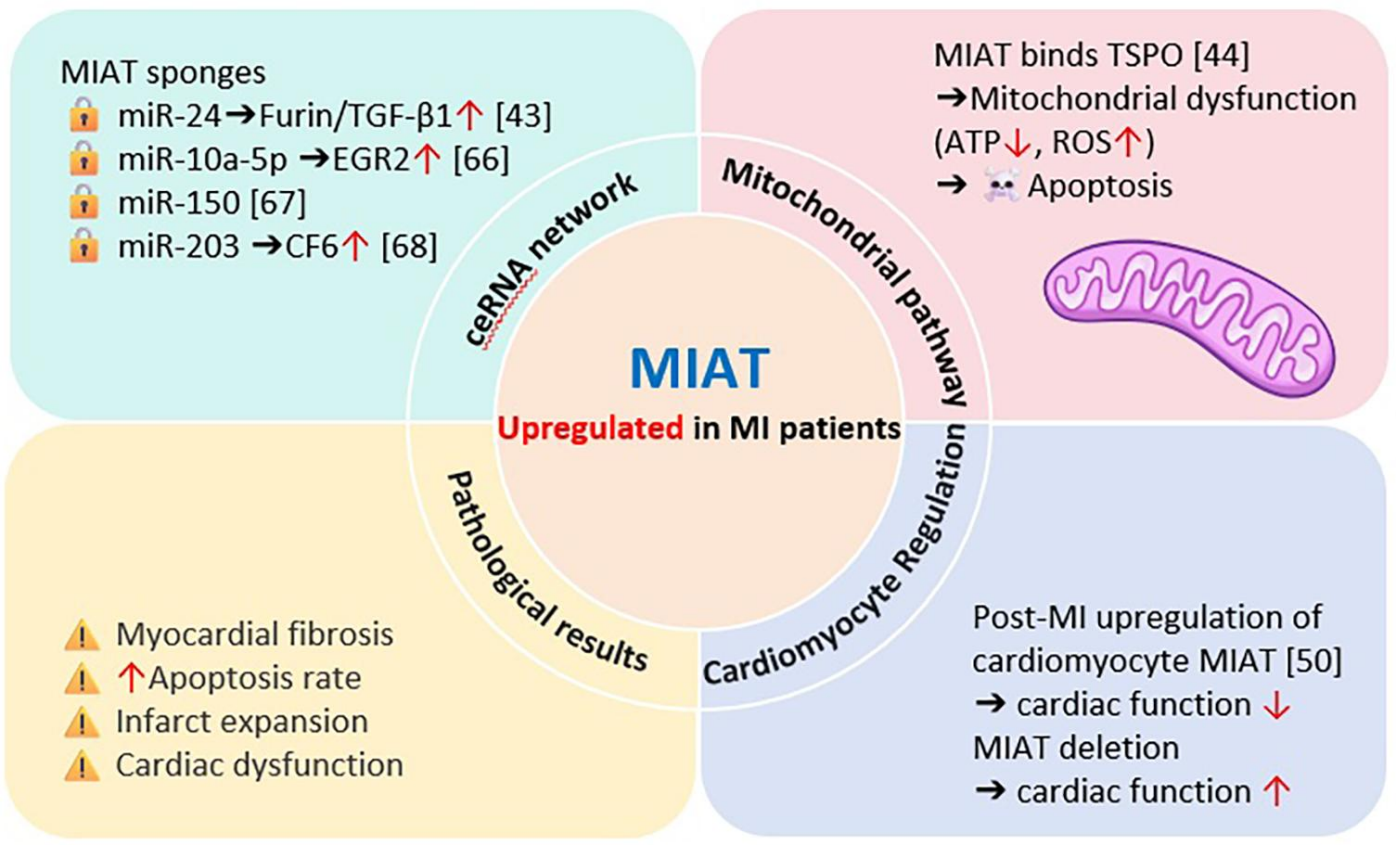
MIAT-mediated molecular pathways and pathological state in myocardial infarction. MIAT acts as a ceRNA by sponging miR-24, miR-10a-5p, miR-150, and miR-203. This leads to upregulation of Furin/TGF-β1, EGR2, and mitochondrial CF6, promoting myocardial fibrosis, apoptosis, and infarct expansion. MIAT also binds TSPO, inducing mitochondrial dysfunction and further apoptosis. Post-MI upregulation of cardiomyocyte MIAT impairs cardiac function, while MIAT deletion relieves myocardial injury and restores cardiac function.

MIAT was initially linked to an increased risk of MI (23, 48). Subsequent studies have demonstrated that MIAT is abnormally overexpressed in the serum, plasma, blood cells, and myocardial tissues of patients with MI, making it a key risk factor for the condition (43, 44, 48). Silencing MIAT in mice with MI results in significant improvements, including a reduction in the MI area, decreased apoptosis rates of cardiomyocytes, and less cardiomyocyte fibrosis, with potential associations to inflammation and metabolism (43, 44, 65). MIAT influences the progression of cardiovascular diseases through multiple molecular pathways, and one proposed mechanism is its potential role as a ceRNA. For instance, MIAT has been reported to interact with miR-24 (43), miR-10a-5p (66), miR-150 (67), and miR-203 (68), promoting the expression of factors such as Furin (43), transforming growth factor-beta1 (TGF-β1) (43), early growth response 2 (EGR2) (66), and mitochondrial coupling factor 6 (CF6) (68), which in turn exacerbate cardiac fibrosis, cardiomyocyte apoptosis, and functional impairment after MI. Additionally, MIAT can bind to proteins, such as the translocator protein (TSPO), leading to mitochondrial dysfunction and cardiomyocyte apoptosis (44). The above-mentioned molecular pathways mediated by MIAT and pathological states in MI are summarized in Figure 2. Research by Azat et al. showed that MIAT expression is low in the hearts of normal rats but markedly elevated in those with AMI (59). Knockout of MIAT significantly reduced cardiomyocyte apoptosis, suggesting that MIAT could serve as a non-invasive marker for early diagnosis or prognostic assessment of MI (58, 59). Furthermore, studies by Hayasaka et al. confirmed that the specific deletion of MIAT in cardiomyocytes markedly improved cardiac function in mice post-MI, clarifying the critical pathological role of cardiomyocyte-derived MIAT in ischemic heart disease (50).

### 5.2 MIAT and myocardial ischemia-reperfusion injury (MI/RI)

The common pathological change of MI/RI is myocardial fibrosis, which is characterized by the excessive deposition of extracellular matrix secreted by activated fibroblasts in the myocardial interstitium (69). Its pathological mechanisms are related to oxidative stress, intracellular calcium overload, metabolic disorders, apoptosis, endoplasmic reticulum stress, mitochondrial dysfunction, autophagy, and ferroptosis (70–72). Chen et al. found that MIAT promotes MI/RI by upregulating the nuclear translocation of NF-κB, inducing the expression of the pro-apoptotic protein PUMA, activating the Caspase-3/Bax pathway, downregulating the expression of Bcl-2, disrupting the balance between pro-apoptotic and anti-apoptotic proteins, exacerbating the apoptosis of cardiomyocytes, and promoting the secretion of pro-inflammatory factors such as TNF-α and IL-6, which intensifies the myocardial inflammatory infiltration (65). Other researches have shown that catechins can downregulate the expression of MIAT by regulating the CREB/MIAT/AKT/GSK-3β pathway, alleviating the apoptosis of cardiomyocytes induced by hypoxia/reoxygenation (73).

### 5.3 MIAT and cardiac hypertrophy

Cardiac hypertrophy is defined as an increase in left ventricular mass caused by the thickening of the left ventricular wall or the enlargement of the chamber diameter (74). The structural manifestations of hypertrophy depend on the type of cardiac injury and can be roughly divided into pressure or volume overload (75). It has become one of the most dangerous risk factors for death in patients with hypertension (76). Regarding the mechanism by which MIAT affects HCM, it is currently believed that there are mechanisms involving the regulation of mitochondrial function and miRNA molecules. Researches have shown that MIAT directly targets the mitochondrial TSPO protein, leading to an increase in mitochondrial membrane permeability, a decrease in membrane potential, and triggering apoptosis of cardiomyocytes and disorders of energy metabolism (43, 44). This research also demonstrated that MIAT promotes the proliferation of fibroblasts and collagen deposition by activating pathways such as TGF-β1/Smad, exacerbating myocardial fibrosis (44). Other researches have found that MIAT competitively binds to miR-29a, relieving its inhibition on fibrosis-related genes (such as collagen synthesis genes) (77), and inhibits the expression of miR-24 (43) and miR-150 (57, 78), upregulates TGF-β1 (43) and Furin (43), accelerates myocardial fibrosis, and thus promotes the formation of cardiac hypertrophy. In a cardiac hypertrophy model, it was found that MIAT binds to the m6A reading protein YTH (YT521-B homology) domain 2 (YTHDF2), regulates the peroxisome proliferator-activated receptor alpha (PPARα)/carnitine palmitoyltransferase 1A (CPT-1A) axis, and promotes abnormal fatty acid metabolism and hypertrophy of cardiomyocytes (79).

### 5.4 MIAT and Ath

**Figure 3:**
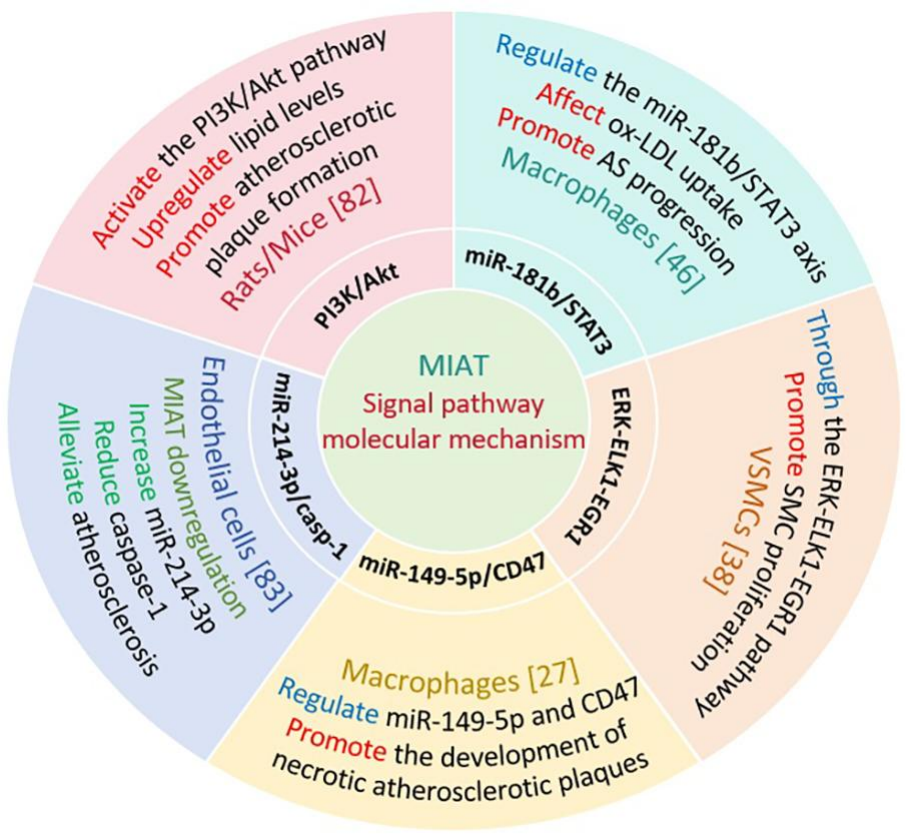
Regulatory mechanisms of MIAT in atherosclerosis. MIAT regulates atherosclerotic progression through multiple signaling pathways in different cell types. In macrophages, MIAT promotes foam cell formation via the miR-181b/STAT3 axis and drives necrotic plaque development by modulating miR-149-5p/CD47. In vascular smooth muscle cells (VSMCs), MIAT enhances proliferation through the ERK-ELK1-EGR1 pathway. In endothelial cells, MIAT downregulates miR-214-3p and Caspase-1 to alleviate atherosclerosis. Additionally, MIAT activates the PI3K/Akt pathway to promote atherosclerotic plaque formation in animal models.

Ath is a vascular disease caused by hyperlipidemia and lipid oxidation, characterized by the formation of intimal plaques (80). These lesions result from an excessive inflammatory and fibrotic hyperplasia response due to endothelial and smooth muscle damage in the arterial wall (81). MIAT plays a pivotal role in the development of Ath by regulating relevant signaling pathways. The regulatory mechanism of MIAT in Ath is illustrated in Figure 3. Sun et al. demonstrated that MIAT upregulates blood lipid levels by activating the phosphatidylinositol 3-kinase (PI3K)/AKT signaling pathway, thereby promomicroting the formation of atherosclerotic plaques and contributing to the progression of Ath (82). Additionally, downregulation of MIAT leads to increased expression of miR-214-3p and reduced expression of Caspase-1 in human aortic endothelial cells, alleviating Ath in ApoE-deficient mice (83). In a mouse model of advanced Ath, MIAT levels were significantly elevated in the serum of patients with vulnerable atherosclerotic plaques and in macrophages within the necrotic core. Mechanistic studies have identified the macrophage MIAT/miR-149-5p/CD47 pathway as a critical factor in the development of necrotic atherosclerotic plaques (27). Furthermore, MIAT can promote the proliferation of smooth muscle cells through the ERK-ELK1-EGR1 pathway (38). Zhong et al. demonstrated that MIAT regulates the miR-181b/STAT3 axis in an Ath cell line induced by the uptake of oxidized low-density lipoprotein (ox-LDL) by macrophages, influencing the pathological progression of Ath (46). In addition to its regulation of signaling pathways, MIAT also participates in the inflammatory response and lipid metabolism, further promoting Ath. Studies have indicated that knocking down MIAT reduces the uptake of ox-LDL, inhibits the activation and translocation of NF-*κ*B, and consequently affects the inflammatory response in Ath (38). This research also highlighted the association between MIAT, lipoprotein (a), and the regulation of ox-LDL, which influences lipid metabolism and participates in the pathological processes of Ath (38).

### 5.5 MIAT and diabetic cardiomyopathy (DCM)

DCM manifests as a metabolic disorder-induced cardiac pathology, marked by progressive myocardial remodeling and functional decline in diabetic patients without hypertension or coronary artery disease (84). Its hallmark feature—systemic lipid metabolism imbalance—impairs myocardial energy homeostasis, driving structural maladaptation and irreversible cardiac dysfunction (85, 86). Research by Zhou et al. has elucidated a novel mechanism whereby MIAT exacerbates DCM by acting as a molecular sponge for miR-22-3p, which in turn upregulates death-associated protein kinase 2 (DAPK2) expression and promotes cardiomyocyte apoptosis (55). Further investigations by Qi et al. revealed that knockdown of MIAT attenuates high glucose-induced cardiomyocyte inflammation, this anti-inflammatory effect is mediated through MIAT's sponging of miR-214-3p (42, 87), which subsequently leads to the downregulation of IL-17, a key pro-inflammatory cytokine implicated in DCM pathogenesis (61). The above studies indicate that MIAT can participate in the pathological process of DCM through the ceRNA mechanism, and targeting MIAT may provide a new intervention strategy for DCM. However, it is noteworthy that current research has not explored effective therapeutic drugs targeting MIAT for anti-DCM treatment. Therefore, regarding future therapeutic strategies for DCM, we propose that on one hand, the therapeutic goal can be achieved by knocking down MIAT using CRISPR technology or antisense oligonucleotides (ASO). On the other hand, existing studies have shown that traditional Chinese medicine components such as berberine (88) and curcumin (89) can exert myocardial protective effects by targeting MIAT. This suggests that intervention strategies targeting MIAT (such as traditional Chinese medicine or compound prescriptions) may have therapeutic potential, but further research is needed to verify the direct interaction between traditional Chinese medicine components and MIAT.

### 5.6 MIAT and stroke

Stroke, also known as cerebrovascular accident, is an acute cerebrovascular disease, which is caused by the interruption of cerebral blood flow or vascular rupture, leading to brain tissue damage, and it has a high disability rate and a high mortality rate, and is divided into ischemic stroke and hemorrhagic stroke (90, 91). In ischemic stroke, elevated MIAT expression has been reported in peripheral blood samples from patients in several clinical cohorts, and survival and multivariate analyses verify that high MIAT level correlates with poor clinical outcomes and serves as an independent prognostic indicator for neurological function recovery and mortality risk (28), though relevant human findings still lack repeated cohort validation. Animal experiments using rat ischemic stroke models and oxygen-glucose deprivation/reoxygenation injured PC12 cells demonstrate that MIAT aggravates ischemic damage by upregulating regulated in development and DNA damage response 1 (REDD1) to promote neuronal autophagy and apoptosis (24). It also interacts with Egl-9 family hypoxia-inducible factor 2 (EGLN2) and reduces MDM2-mediated K48 polyubiquitination to stabilize EGLN2 protein, and the MIAT-EGLN2 axis disrupts neuronal redox homeostasis and exacerbates cerebral ischemia-reperfusion injury (45). Beyond neurons, MIAT has been suggested to competitively bind miR-204-5p, potentially leading to upregulation of high-mobility group box 1 (HMGB1) expression, thereby triggering cerebrovascular endothelial cell damage and impairing vascular stability after ischemia (92). Moreover, MIAT has been shown to regulate neuronal injury and inflammation through a miR-874-3p/IL-1B-dependent pathway (62). In summary, MIAT exerts differentiated regulatory effects on diverse cerebral cell types, inducing apoptosis in neurons, destroying barrier function of endothelial cells and mediating glial inflammatory responses, and the acute attack and early recovery phases are regarded as the optimal therapeutic window for targeted MIAT intervention. As for hemorrhagic stroke, clinical investigations have reported abnormal MIAT upregulation in patient peripheral specimens, suggesting its potential as a disease biomarker (63). Relevant research also confirmed MIAT modulates vascular lesion progression by regulating ectodermal-neural cortex 1 (ENC1) expression via myelocytomatosis oncogene (MYC) mediation, further driving cerebral vascular damage and bleeding risk (93). In conclusion, silencing MIAT effectively alleviates hematoma swelling and nerve functional impairment in animal models. Current evidence is still insufficient in large-sample human validation, and its cell-specific functions and stage-dependent variation remain to be clarified. Early acute intervention targeting MIAT is conducive to restraining secondary neurological damage.

The mechanisms of action of MIAT in different cardiovascular and cerebrovascular diseases are summarized in Table 2. To date, mechanisms marked with asterisks (*) have been validated in human serum or blood samples from available clinical studies, providing supportive clinical evidence for their pathological relevance in human cardio-cerebrovascular diseases. Explanation of evidence tiers in Table 2: Level I: Expression correlation only. Level II: *in vitro* knockdown/overexpression without rescue. Level III: *in vivo* genetic manipulation showing functional phenotype. Level IV: Mechanistic rescue plus direct binding evidence.

**Table 2:** The mechanism of MIAT in cardiovascular and cerebrovascular diseases.

Disease	Molecular mechanism (miRNA/axis)	Evidence level	References
Myocardial infarction	miR-24/TGF-β1/Furin	Level IV	(43, 44, 66–68)
	miR-150	Level II–III	
	miR-10a-5p/EGR2	Level IV	
	miR-203/CF6	Level III	
	Binding to TSPO	Level III–IV	
Myocardial Ischemia -Reperfusion Injury	NF-*κ*B/PUMA	Level III	(65, 94)
	miR-181a-5p/JAK2/STAT3	Level II–III	
Cardiac hypertrophy	miR-29a-3p*	Level IV	(57, 77, 79)
	miR-150/P300	Level II	
	Binding to YTHDF2	Level II	
Atherosclerosis	miR-214-3p/Caspase-1	Level II–III	(27, 46, 82, 83)
	miR-149-5p/CD47	Level III–IV	
	miR-181b/STAT3	Level IV	
	Activating PI3K/Akt	Level III	
Diabetic cardiomyopathy	miR-22-3p/DAPK2	Level IV	(55, 61)
	miR-214-3p/IL-17*	Level II–III	
Stroke	miR-874-3p/IL1B*	Level II–III	(24, 45, 62, 92, 93)
	miR-204-5p/HMGB1	Level II–III	
	EGLN2 protein interaction	Level III–IV	
	Upregulating REDD1/mTOR	Level III–IV	
	MYC/ENC1	Level II–III	

## 6 MIAT as a biomarker and potential therapeutic target

Biomarkers, as quantifiable biological indicators, offer a non-invasive or minimally invasive means to detect and dynamically reflect the body's physiological state, disease progression, and treatment response, providing valuable pathological insights even during the asymptomatic stage (95, 96). Their core value spans multiple areas, including early disease screening (44, 58, 59, 97), risk stratification (98, 99), treatment monitoring (44, 58, 59, 70, 79), and new drug development (96, 100). As reported in several studies, MIAT exhibits relative stability in serum exosomes under standardized sample processing and quantitative real-time reverse-transcription polymerase chain reactio (qRT-PCR) detection conditions, and its expression is markedly altered in gastric cancer patients relative to gastric adenoma patients and healthy controls (101, 102). The maturation of isolation and identification technologies, based on exosomal surface markers such as TSG101, CD63, and Flotillin-1, combined with standardized detection methods like qRT-PCR, provides a reliable methodological foundation for the clinical translation of MIAT (43, 59, 103, 104). Current clinical studies suggest MIAT possesses promising diagnostic potential in cardiovascular diseases. In single-center retrospective cohort, its diagnostic performance shows comparable trends with cardiac troponin T (cTnT, the established gold-standard marker), and may present potential advantages for early disease identification in limited clinical observations (59). Nevertheless, the finding is derived from single-center, small-sample retrospective cohorts, without large-scale prospective validation or unified pre-analytical and detection standards. Further investigations focusing on dynamic MIAT expression changes and disease prognosis including recurrence risk and mortality will help consolidate its value as a prognostic biomarker (38, 105).

Regarding therapeutic target development, MIAT exhibits potential for cross-disease regulation; however, its translational application is hindered by several biological challenges. Mechanistic studies have shown that MIAT is involved in pathological processes through complex molecular networks. For instance, in a neuropathic pain model, MIAT upregulates bone morphogenetic protein and activin membrane-bound inhibitor expression by adsorbing miR-362-3p, thereby activating the TGF-β signaling pathway to promote disease progression (106). This ceRNA regulatory model suggests that targeting MIAT alone could disrupt the cascade reactions mediated by its interacting molecules (e.g., miRNAs, protein complexes), emphasizing the need for a systematic analysis of its tissue-specific interaction network to avoid off-target effects. Furthermore, the functional heterogeneity of MIAT across different diseases adds complexity to therapeutic strategy design. For instance, MIAT inhibitors targeting myocardial hypertrophy must be rigorously evaluated for potential effects on other pathological contexts where MIAT may exert distinct, even opposing, roles.

In the development of therapeutic targets for lncRNA, there are currently three common targeted knockdown strategies, namely ASO, siRNA, and CRISPR-Cas9 gene editing technology. Among them, all three therapeutic strategies targeting MIAT have been explored in cardiovascular and cerebrovascular diseases. For example, Park et al. used a cardiomyocyte-specific CRISPR-dCas9 system to silence MIAT, and the cardiac function of a mouse model of ischemic heart disease was significantly improved (107). In addition, miRNA mimics or inhibitors provide an indirect regulatory approach by modulating key miRNAs that are “sponged” by MIAT [such as miR-133a-3p (17, 60) and miR-214-3p (61, 83)] to influence MIAT's functional network.

Although these strategies show great potential, their clinical translation still needs to overcome key challenges such as tissue-specific delivery efficiency and potential off-target effects. For instance, the functional heterogeneity of MIAT in different diseases increases the complexity of therapeutic strategy design. MIAT inhibitors developed for myocardial hypertrophy need to be strictly evaluated for potential off-target effects on other pathological processes involving MIAT, such as tumorigenesis and neurodegenerative diseases. Therefore, constructing disease-specific delivery systems and developing spatiotemporally precise regulation technologies will become key directions for future research. The situation of MIAT as a biomarker and therapeutic target is summarized in Table 3.

**Table 3:** MIAT as a biomarker and therapeutic target.

Application	Advantages	Challenges	References
Diagnostic biomarker	Relative stability in serum exosomes Comparable sensitivity to cTnT in AMI cohorts	Limited sample sizes in existing studies Lack of unified standardized detection protocols	(97, 101, 102)
Prognostic marker	Expression levels correlate with disease staging Serves as an independent predictor of mortality risk	Expression heterogeneity across different diseases Unbalanced cost-benefit for routine clinical use	(38, 58, 59, 105)
Therapeutic target	Regulates cross-disease pathways Feasible silencing via CRISPR, ASO and siRNA strategies	Off-target risks due to complex multi-pathway networks Poor tissue-specific delivery efficiency *in vivo*	(107)

## 7 Summary and prospect

As a pivotal lncRNA, MIAT demonstrates dual potential in the diagnosis and treatment of cardiovascular and cerebrovascular diseases.In current clinical detection systems, serum-based MIAT measurement exhibits comparable diagnostic sensitivity to established biomarkers such as cTnT in limited cohort studies (58, 59). Mechanistic studies have shown that MIAT influences multiple pathological processes: in ischemic stroke, it exacerbates nerve cell autophagy/apoptosis via the REDD1-mTOR axis (24); while in AS, it activates the NLRP3 inflammasome through the miR-214-3p/Caspase-1 pathway, accelerating plaque progression (83). However, the full complexity of its molecular network, such as ceRNA regulation and three-dimensional genomic interactions, remains to be fully understood. Additionally, the absence of tissue-specific delivery systems and multi-target synergistic treatment strategies raises concerns about off-target effects in current intervention methods (108). Regarding clinical translation, despite the establishment of a detection system based on qRT-PCR, challenges such as limited sample sizes (*n* < 1,000), insufficient detection standardization, and cost-benefit imbalances hinder the widespread application of MIAT as a biomarker (109, 110).

To address the above translational challenges, future research should prioritize feasible, MIAT-oriented preclinical optimizations and near-term clinical verifications. Mechanistically, the combination of single-cell spatial transcriptomics and conditional gene editing technologies helps elucidate the cell-specific, dynamic regulatory networks of MIAT within the pathological microenvironment of cardio-cerebrovascular diseases (111). The application of CRISPR-dCas9 epigenetic silencing tools and endothelium-targeted nanocarriers significantly improves the targeting accuracy and intervention efficiency of MIAT modulation and minimizes non-specific biological effects (112, 113). Notably, tailored tissue-specific delivery strategies for cardiomyocytes, vascular endothelial cells, and macrophages need to be optimized separately, considering the distinct pathogenic roles of MIAT in different cellular subtypes. Of particular concern is the inherent on-target safety risk: since MIAT also participates in physiological and pathological processes in the central nervous system and tumor tissues, global suppression of MIAT may trigger unexpected off-organ adverse effects, requiring rigorous preclinical evaluation of organ-specific safety profiles. For translational validation, developing stable and sensitive target engagement biomarkers is essential to monitor effective MIAT inhibition and downstream pathway changes, which serves as a core criterion for evaluating pharmacological responses. At the current research stage, large-scale biobank construction, machine learning-based batch validation, and Phase III clinical trials are still premature and impractical. Instead, early and reversible cardio-cerebrovascular inflammatory and ischemic lesions with consistent MIAT upregulation represent the most appropriate and feasible indications for preliminary translational exploration. Moreover, standardized optimization of sample collection, processing, and detection protocols will improve the repeatability and reliability of MIAT-based biomarker detection, laying a solid and progressive foundation for the gradual clinical translation of MIAT-targeted therapeutic strategies.

## 8 Literature search methodology

This work is a narrative review. We searched the PubMed and Web of Science databases for relevant literature using the keywords: MIAT, lncRNA, myocardial infarction-associated transcript, cardiovascular disease, cerebrovascular disease, mechanism, biomarker, therapeutic target. The search was limited to English-language articles published from database inception to May 2025. Both original research articles, valuable review papers and conference abstracts discussing MIAT's biological functions, disease associations, and translational potential were included. No formal quality assessment was performed, and all eligible studies were synthesized narratively to provide an up-to-date overview of MIAT research in cardiovascular and cerebrovascular diseases.
